# HOW TO GET AN NIA GRANT

**DOI:** 10.1093/geroni/igz038.1508

**Published:** 2019-11-08

**Authors:** Robin A Barr

**Affiliations:** National Institute on Aging, National Institutes of Health, Bethesda, Maryland, United States

## Abstract

Dr. Barr will provide an overview of the various funding mechanisms supported by NIA, with emphasis on mechanisms that are most appropriate for early career scientists.

